# Supplementary material for: Highly Sensitive Detection for Mercury Ions Using Graphene Oxide (GO) Sensors
Source: Micromachines (Basel). 2021 Sep 2;12(9):1070. doi: 10.3390/mi12091070 (PMC8470607; doi:10.3390/mi12091070)
Supplement: Supplementary file 1 [file micromachines-12-01070-s001.zip › micromachines-1305267-supplementary.pdf]

micromachines

Highly Sensitive Detection for Mercury Ions Using Graphene Oxide (GO) Sensors

Li Gao, Haixia Shi, Raoqi Li, Cheng Liu, Jia Cheng and Lei Liu

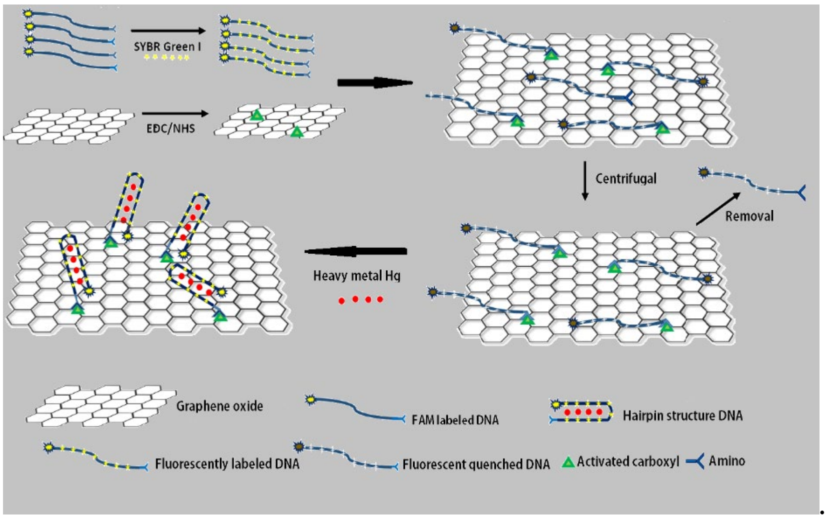

Figure S1. Scheme for GO-DNA sensor for detection of Hg^{2+}.

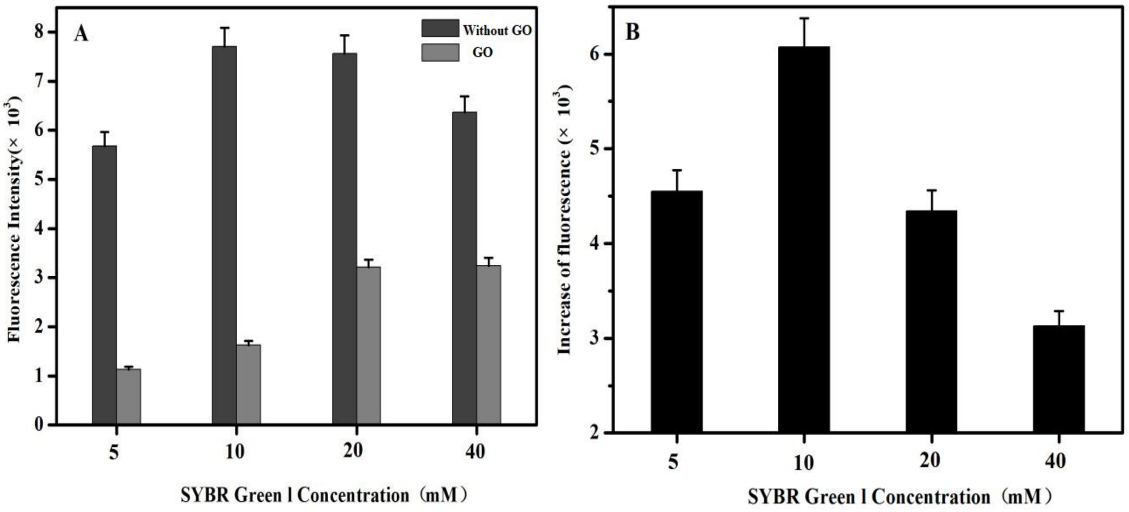

Figure S2. (**A**) Effect of GO on the concentration of SYBR Green I modified aptamer in different concentrations; (**B**) Fluorescence change values after adding GO to different concentrations of SYBR Green I modified aptamer.

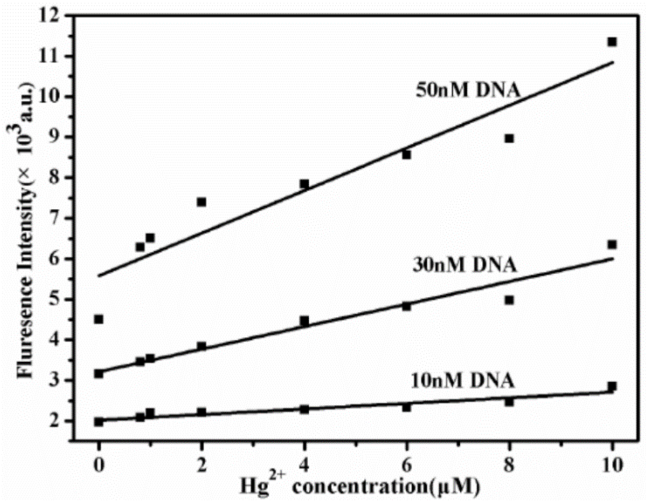

Figure S3. Different concentrations of DNA were constructed with different biosensors. GO concentration was 10 μg/mL, the concentration of DNA in turn from top to bottom was 10 nM, 30 nM, 50 nM.

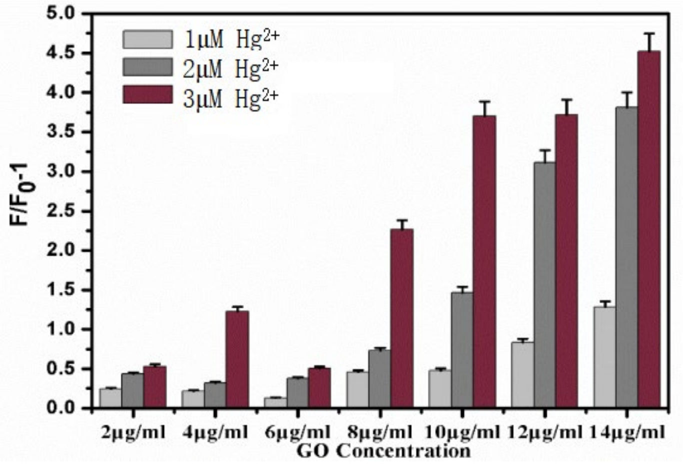

Figure S4. The effect on the fluorescence of DNA modified with Green I in different concentrations of GO. GO concentration from left to right was 2 μg/mL, 4 μg/mL, 6 μg/mL, 8 μg /mL, 10 μg/mL, 12 μg/mL and 14 μg/mL.

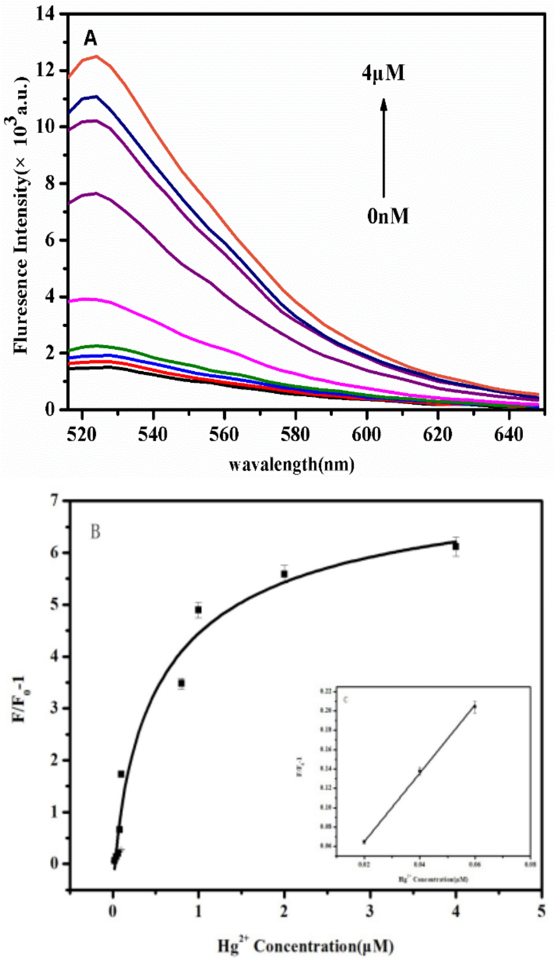

Figure S5. Detection of Hg^{2+} using physical adsorption of DNA. (A)The fluorescence intensity of GO-DNA sensor after adding different concentrations of Hg^{2+}.(B) The value of F/F_0-1 after adding different concentrations of heavy metal mercury. F and F_0 were the fluorescence intensity of after adding Hg^{2+},and before adding Hg^{2+}. (C)The illustration showed a linear relationship between low concentrations of Hg^{2+} and F/F_0-1.

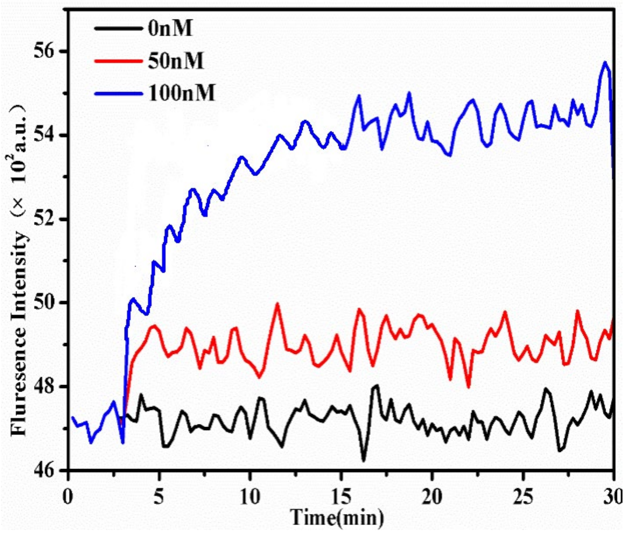

Figure S6. The fluorescence intensity of FAM-modified GO-DNA probe at different times.
